# Effectiveness and safety of coenzyme Q10 in people with heart failure with preserved ejection fraction (HFpEF): a systematic review and meta-analysis

**DOI:** 10.1136/bmjopen-2025-116033

**Published:** 2026-10-13

**Authors:** Ebadur Rahman, Katie E Webster, Angus K Nightingale, Maria Pufulete

**Affiliations:** 1 University of Bristol, Bristol, UK; 2 NIHR Bristol Evidence Synthesis Group, Population Health Sciences, University of Bristol, Bristol, UK

**Keywords:** Heart failure, CLINICAL PHARMACOLOGY, Adult cardiology, Systematic Review, Meta-Analysis

## Abstract

**Abstract:**

**Objectives:**

Coenzyme Q10 (CoQ10) supplementation has previously shown efficacy in improving symptoms and outcomes for people with heart failure with reduced ejection fraction. This review aims to assess whether CoQ10 may also be effective and safe for those with heart failure with preserved ejection fraction (HFpEF).

**Design:**

Systematic review and meta-analysis of randomised controlled trials.

**Data sources:**

We searched databases including Embase, MEDLINE, CENTRAL, Web of Science, CINAHL, AMED, ClinicalTrials.gov and the ISRCTN registry on 25 June 2025.

**Eligibility criteria:**

We included randomised controlled trials (RCTs) enrolling adults (≥18 years) with HFpEF, defined as an ejection fraction of ≥50%. Eligible studies compared any CoQ10 regimen with placebo or standard care.

**Data extraction and synthesis:**

Two reviewers independently screened studies and extracted data. Risk of bias was assessed using RoB2 and the certainty of evidence using Grading of Recommendations Assessment, Development and Evaluation (GRADE). We conducted random-effects pairwise meta-analysis where possible, using the RevMan V.5.4 software. Synthesis without meta-analysis was conducted for other outcomes.

**Results:**

Three RCTs (264 participants) with a treatment duration of 8–24 weeks were included. The effect of CoQ10 on mortality in individuals with HFpEF was very uncertain (2/101 participants receiving CoQ10 vs 0/105 participants receiving placebo). No cardiovascular deaths occurred in either group (206 participants, very low certainty evidence). The effect of CoQ10 on hospitalisation due to heart failure was very uncertain (1/19 participants receiving CoQ10 vs 0/20 participants receiving placebo). Evidence on quality of life was mixed and also considered very uncertain, with one study indicating benefit from CoQ10 (mean difference (MD) 22.41 points on the Kansas City Cardiomyopathy Questionnaire, 95% CI 12.18 to 32.64, 73 participants) and another indicating no benefit (MD −8.52 points, 95% CI −19.20 to 2.16, 66 participants).

**Conclusion:**

Current evidence is insufficient to determine whether CoQ10 improves outcomes in HFpEF. Larger, high-quality and adequately powered RCTs using consistent diagnostic criteria are needed before CoQ10 can be recommended in clinical practice.

**PROSPERO registration number:**

CRD420251027238.

**STRENGTHS AND LIMITATIONS OF THIS STUDY**

- This review was conducted in accordance with the Preferred Reporting Items for Systematic Reviews and Meta-Analyses (PRISMA) 2020 guidance and followed a prospectively registered protocol.
- No language restrictions were used, maximising identification of relevant primary studies.
- Quantitative synthesis was not possible for many outcomes due to heterogeneity in intervention formulations, dosing regimens, cointerventions and outcome measures.

## Introduction

 Heart failure is a chronic, progressive syndrome in which the heart is unable to pump enough blood to meet the body’s metabolic needs.^1^ The condition is characterised by key symptoms such as breathlessness, fatigue and reduced exercise tolerance.^2^ Globally, more than 64 million people are affected^3^ and the prevalence continues to rise alongside other common conditions such as hypertension, obesity and diabetes.^4^ The disease rarely follows a steady trajectory; it is characterised by a relapsing-remitting pattern with periods of stability interrupted by acute decompensation.^5^ Over time, these recurrent episodes place increasing strain on the heart muscle.^6^

Left ventricular ejection fraction (LVEF) is commonly used to classify heart failure into three main phenotypes. Heart failure with reduced ejection fraction (HFrEF) is defined as an EF<40% while heart failure with preserved ejection fraction (HFpEF) is defined as an EF ≥50%. People with an EF that falls outside these thresholds (EF 40%–49%) are classified as HF with mildly reduced EF, and guidelines recommend treatment as for HFrEF.^7^ HFpEF now accounts for around half of all HF cases.^8 9^ Despite this, there are few disease-modifying pharmacotherapies for HFpEF.^10–13^ While therapeutic options for HFrEF expanded considerably in recent years, the management of HFpEF involves controlling symptoms rather than preventing progressive damage to the heart.^14^ Current treatment focuses on optimising blood pressure, managing comorbidities and using diuretics to manage congestion.^15^

The pathophysiology of HFpEF is complex and multifactorial. Proposed mechanisms include microvascular endothelial dysfunction, impaired left-ventricular relaxation, increased myocardial stiffness and systemic inflammation.^16^ A key driver of HFpEF appears to be impaired myocardial energetics. Mitochondrial dysfunction and oxidative stress create an energy deficit, reducing ATP generation and disrupting diastolic filling.^17^

Coenzyme Q10 (CoQ10), also known as ubiquinone, is a naturally occurring, fat-soluble vitamin-like substance. It functions as an electron carrier in the oxidative phosphorylation pathway, supporting ATP synthesis.^18^ CoQ10 also has antioxidant properties, protecting cellular membranes from oxidative damage.^19^ Healthy people make sufficient CoQ10 in their body, but people with HF often have low tissue levels of CoQ10, which inversely correlate with disease severity.^20^ Therefore, CoQ10 supplementation has been proposed as a biologically plausible approach to improve myocardial energetics in people with HFrEF and HFpEF.

Several recent randomised controlled trials (RCTs)—notably the Q-SYMBIO^21^ study—have reported improved patient outcomes following CoQ10 supplementation in patients with HFrEF. These findings provide a rationale for investigating whether CoQ10 also improves outcomes in people with HFpEF.

Although several systematic reviews and meta-analyses have evaluated the effectiveness and safety of CoQ10 for HF, most have pooled participants across HF phenotypes. The majority of studies focus on HFrEF, with minimal representation of individuals with HFpEF and without subgroup analysis. Two recent reviews have specifically evaluated CoQ10 use in people with HF. Claxton *et al*^22^ conducted a systematic review of CoQ10, but restricted their scope to HFrEF. This review concluded that the existing evidence base was insufficiently robust. More recently, a meta-analysis conducted by Xu *et al*^23^ pooled data across HF phenotypes, with the vast majority focusing on HFrEF. This review indicated some benefit to the use of CoQ10, but no subgroup analysis was conducted to determine whether the efficacy of treatment may vary across clinical phenotypes.

The HFpEF population is more heterogeneous and has an older age profile. Given the distinct pathophysiology and treatment response of HFpEF compared with HFrEF, a focused analysis specific to HFpEF is appropriate. This review aims to identify, appraise and synthesise evidence from RCTs evaluating the efficacy and safety of CoQ10 supplementation in adults with HFpEF exclusively.

### Objective

To assess the effectiveness and safety of CoQ10 in adults with HFpEF.

## Methods

This review was registered prospectively in PROSPERO (CRD420251027238) and reported according to the Preferred Reporting Items for Systematic Reviews and Meta-Analyses (PRISMA) 2020 guidelines. The review protocol is also available via the PROSPERO database.^24^

### Eligibility criteria

We included randomised controlled trials only, including parallel, cluster and cross-over designs. For cross-over trials, we planned to include only the first phase of the study (prior to participant cross-over). Eligible participants were adults (aged ≥18 years) diagnosed with HFpEF, defined by LVEF≥50% plus clinical signs or symptoms of HF, according to study criteria. Studies including people with both reduced and preserved EF HF were eligible if ≥80% of participants had HFpEF or where subgroup data were available specifically for those with HFpEF.

We excluded studies that were conducted exclusively in people with HFrEF. We also excluded studies conducted in people with an acute exacerbation of HF (defined as a rapid onset and acute worsening of signs and symptoms associated with HF, typically leading to hospitalisation). Any CoQ10 formulation (ubiquinol/ubiquinone), dose and duration, administered with/without antioxidant co-supplementation (eg, D-ribose, selenium, vitamin E) were included, where CoQ10 was compared with placebo or no intervention. We anticipated that participants in both the intervention and comparator arms would receive standard care.

### Outcome measurement

Our primary outcomes were all-cause mortality, cardiovascular mortality, hospitalisation due to HF and quality of life. Secondary outcomes were hospitalisation due to any cause, New York Heart Association (NYHA) class, exercise capacity*,* blood natriuretic peptide levels and adverse events.

### Information sources and search strategy

We were aware of a relatively recent Cochrane review that considered the use of CoQ10 for all individuals with HF.^25^ First, we searched the reference lists of the Cochrane review (both included and excluded studies) to identify articles that should be assessed in full text. This is the step referred to in the PRISMA diagram as ‘identification of studies via other methods’. Then we ran an updated search (from 16 October 2020 onwards—the date of the last search for the Cochrane review) to capture additional articles published since the original searches in the Cochrane review (referred to as ‘identification of studies via databases and registers’). Electronic searches were conducted across MEDLINE (Ovid), CENTRAL (Cochrane Library), Web of Science, CINAHL, AMED, ISRCTN^26^ and ClinicalTrials.gov.^27^ The full search strategies for all databases and registers are available as a supplementary file (see online supplemental file).

We also hand searched the reference lists of two recent systematic reviews on CoQ10 for HF.^23 25^ To ensure that all relevant references were captured, we checked the full text for all articles included in these reviews as well as all articles that were cited as excluded studies.

A breakdown of records retrieved, screened and included is shown in figure 1 (PRISMA flow diagram).

**Figure 1:**
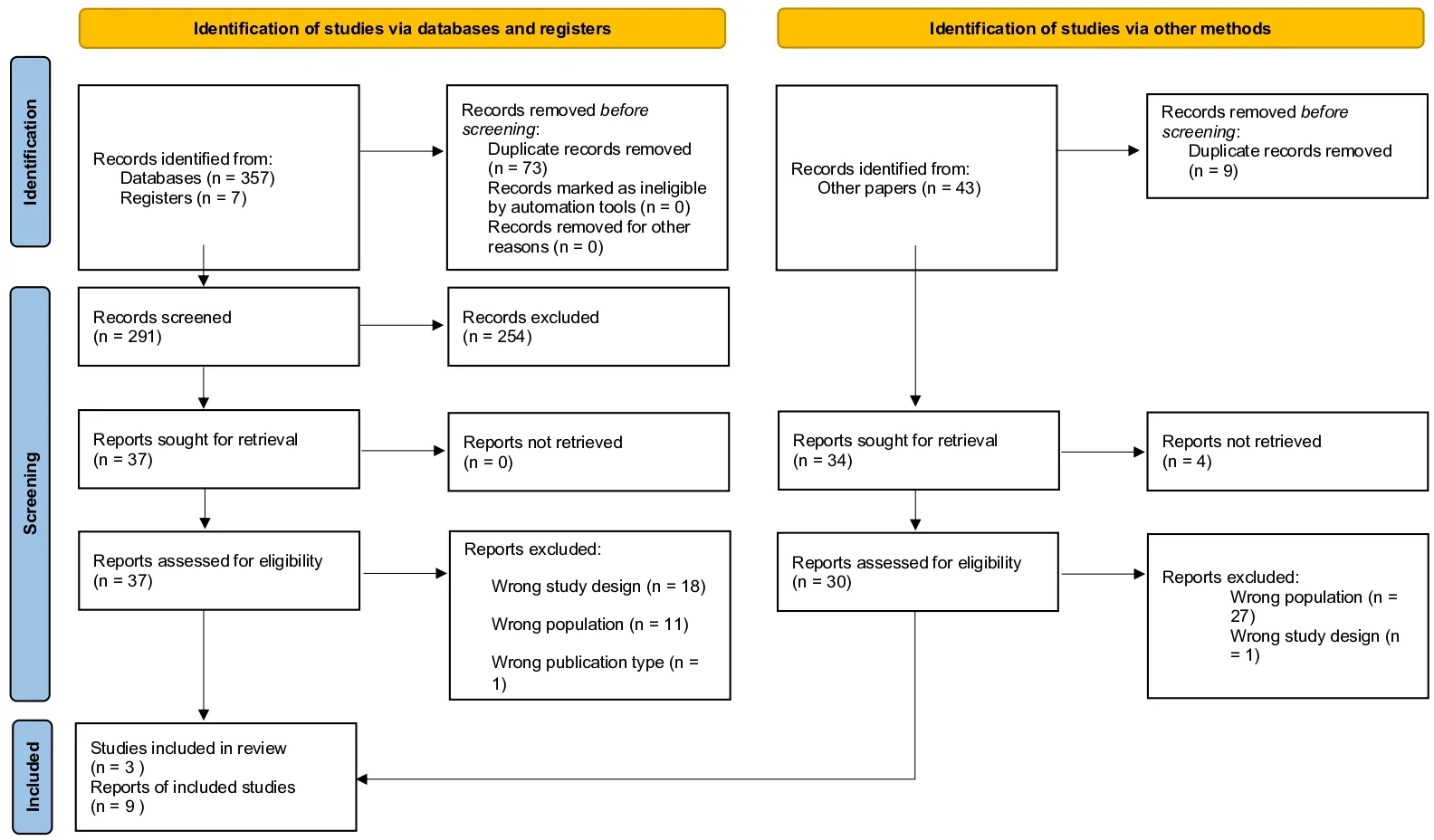
Preferred Reporting Items for Systematic Reviews and Meta-Analyses (PRISMA) flow diagram summarising the searching, screening, records included/excluded with reasons and included studies.

### Study selection and data extraction

The titles and abstracts of records retrieved through bibliographic and hand searches were screened on Rayyan^28^ by two reviewers independently (ER, KEW). Conflicts were resolved by discussion. The full text of any potentially relevant records was obtained. Full texts were also screened independently by two reviewers (ER, KEW). Conflicts were resolved through discussion and consensus, or by recourse to a third reviewer. The PRISMA flow diagram (figure 1) documents the selection process.

A piloted form was used to standardise extraction across all included studies. Data extraction was completed by one reviewer (ER) and independently checked for accuracy by a second (KEW). We collected: study design/setting; definition of HFpEF; eligibility criteria; sample size; intervention and comparator details (including formulation, dose, duration and any cointerventions); outcomes (including time of assessment and number assessed).

### Risk of bias assessment

Risk of bias was assessed for each primary outcome with the Risk of Bias 2 tool (RoB2).^29^ Risk of bias was assessed by a single reviewer (ER) and discussed with a second reviewer where necessary (KEW).

### Certainty assessment

The Grading of Recommendations Assessment, Development and Evaluation (GRADE) approach was used to evaluate the certainty of evidence for each primary outcome.

### Data synthesis methods

Outcomes were considered for quantitative synthesis only when ≥2 study arms reported sufficient comparable data, and the contributing studies were similar in terms of participants, interventions, comparators and outcomes.

We extracted information on the number of participants assessed, and either the number of events (for dichotomous data), or the mean value and SD for both the intervention and comparator groups. When required, SDs were calculated from reported CIs or SEs. Where necessary, effect estimates were calculated based on data reported in the studies. We summarised study characteristics and results in structured tables using Microsoft Excel.

Where ≥2 sufficiently comparable studies reported an outcome, we conducted random-effects meta-analyses with the Mantel-Haenszel method using Cochrane RevMan software^30^ and quantified statistical heterogeneity using the I^2^ statistic.^31^ However, due to the extreme scarcity of events for many of our outcomes, we considered that any statistical synthesis may produce unreliable estimates. Instead, we present a synthesis without meta-analysis for some outcomes, due to the scarcity of data or because we considered it inappropriate to present a pooled estimate (reasons described in the text).

One 2×2 factorial study^29^ reported outcomes as contrast data between groups, and one contrast of interest was not reported (CoQ10 plus D-ribose vs D-ribose alone). The mean effect for this comparison was estimable using data reported for other group contrasts. However, to provide an estimate of the variance for this contrast, we calculated the mean variance across all other reported contrasts for the outcome. CIs estimated using these imputed data are identified throughout the Results section to indicate the consequent uncertainty.

We planned to conduct subgroup analyses considering the effect of age, sex and comorbidities, given sufficient data and compatible comparisons.

According to our protocol, we excluded data assessed at high risk of bias from the primary synthesis, but planned to conduct sensitivity analyses including all data, regardless of risk of bias.

### Patient and public involvement

Patients and members of the public were not involved at any stage of this review.

## Results

A total of 325 unique articles were identified from our searches. The full text of 71 articles was assessed, including 34 records identified from an existing Cochrane review^25^ (of CoQ10 for all types of HF), and an additional 37 records retrieved by our new searches. Three studies were included in the review (see figure 1).

### Study characteristics

The three RCTs were small in size (30–153 participants) and short in duration (8–24 weeks). All studies enrolled adults with HFpEF (LVEF≥50%). All investigated oral CoQ10 supplementation compared with either placebo or no intervention (see table 1).

**Table 1:** Characteristics of included studies

Study	Country	Sample (n) (experimental/control)	Age (mean±SD)	Baseline LVEF (%) (mean±SD)	Baseline NYHA class	Intervention*	Control*	Follow-up
Pierce *et al*†^36^	USA	39/39	69‡	51.89±5.68/51.04±7.43	II–III	CoQ10 600 mg/day	Placebo	12 weeks
Pierce *et al*†^36^	USA	37/38	69‡	51.28±4.76/50.79±4.38	II–III	CoQ10 600 mg/day+D-ribose 15 g/day	Placebo+D-ribose	12 weeks
Samuel *et al*^32^	Israel	19/20	76±8.97/74.8±9.99	59.1±6.1/59.3±6.1	II–III	CoQ10 300 mg (administered as Ubiquinone (100 mg three times/day))	Placebo capsules	4 months
Sobirin *et al*^33^	Indonesia	15/15	64±10/61±7	55±4/58±8	II–III	CoQ10 300 mg (administered as Ubiquinone (100 mg three times/day))	Routine HF therapy alone	30 days

* All trials provided standard therapy in addition to the intervention/control.

† This single study provides data for two separate comparisons (and so each comparison is treated individually).

‡ SDs of age were not reported for these trials.

CoQ10, coenzyme Q10; HF, heart failure; LVEF, left ventricular ejection fraction; NYHA, New York Heart Association.

Samuel *et al*^32^ (UK) was a double-blind, placebo-controlled trial of CoQ10 (administered as Ubiquinone) 100 mg three times per day for 24 weeks alongside standard HF therapy with a total of 39 participants. The control group received placebo alongside standard HF therapy. Outcomes were all-cause and cardiovascular mortality, hospitalisation due to HF, hospitalisation due to any cause, NT-proBNP and adverse events.

Sobirin *et al*^33^ (Indonesia) was an open-label RCT of CoQ10 (administered as Ubiquinone) 300 mg per day for 6 weeks in 30 participants. The control group received standard HF therapy alone. Outcomes included all-cause and cardiovascular mortality, and adverse events.

Pierce *et al*^34^ (USA) was a four-arm, double-blind RCT with a factorial design, assessing both CoQ10 (administered as Ubiquinol) and D-ribose. CoQ10 was administered orally at 400 mg per day for 12 weeks, either alone or with D-ribose (15 g/day). For the purposes of our analysis, we provide a comparison of those who received CoQ10 versus placebo and those who received CoQ10 plus D-ribose versus those who received D-ribose alone. We considered that the use of D-ribose as a cointervention in two groups may act as an effect modifier for CoQ10, and consequently chose not to pool efficacy data from these two comparisons. Reported outcomes included mortality, cardiovascular mortality, quality of life (Kansas City Cardiomyopathy Questionnaire, KCCQ), 6-minute walk test, BNP and adverse events (these were listed as ‘serious adverse events’). Additional outcomes were reported in the trial registry site.^35^

### Risk of bias and certainty of evidence

We had some concerns regarding the potential for bias with all of the included data (see table 2). For two studies,^32 33^ this was due to incomplete reporting of methods used for allocation concealment and lack of a published prespecified analysis plan. A published protocol^36^ was identified for Pierce *et al*.^34^ However, some data were missing, and an intention-to-treat analysis was not conducted for one comparison. No data for our primary outcomes were considered to be at high risk of bias; therefore, all evidence was included in the synthesis.

**Table 2:** Risk of bias summary

Study	Outcome	D1*	D2†	D3‡	D4§	D5¶	Overall	Description of concerns/comments
Pierce 2018a^36^	All-cause mortality							Some data were missing for this outcome (5/78 participants; 6.4%); however, this was not considered likely to depend on the true value
	Cardiovascular mortality							As above
	Quality of life							As above
Pierce 2018b^36^	All-cause mortality							For this comparison, data for two participants were excluded due to non-adherence to the protocol; however, the impact on the results is not likely to be significant. Some data were missing for this outcome (9/75 participants; 12%); however, this was not considered likely to depend on the true value
	Cardiovascular mortality							As above
	Quality of life							As above
Samuel 2022^32^	All-cause mortality							Computerised randomisation was performed, but there is no description of how/whether group allocation was concealed. No prespecified analysis plan was available
	Cardiovascular mortality							As above
	Hospitalisation due to heart failure							As above
Sobirin 2019^33^	All-cause mortality							Computerised randomisation was performed, but there is no description of how/whether group allocation was concealed. No prespecified analysis plan was available
	Cardiovascular mortality							As above

Colour coding: yellow=some concerns regarding risk of bias; green=low risk of bias.

Pierce 2018a: comparison of CoQ10 with placebo, Pierce 2018b: comparison of CoQ10 plus D-ribose with D-ribose.

* Randomisation process

† Deviations from the intended outcome.

‡ Missing outcome data.

§ Measurement of the outcome.

¶ Selection of the reported result.

Our assessments of the certainty of the evidence for our primary outcomes are reported in table 3.

**Table 3:** Summary of findings

Certainty assessment							Patients (n)		Effect	Certainty
Studies (n)	Study design	Risk of bias	Inconsistency	Indirectness	Imprecision	Other considerations	CoQ10	No treatment/placebo		
All-cause mortality										
4	Randomised trials	Serious*	Not serious	Not serious	Very serious†	None	2/101 (2.0%)	0/105 (0.0%)	Two studies reported one death each, both in people receiving CoQ10	⨁◯◯◯ Very low*†
Cardiovascular mortality										
4	Randomised trials	Serious*	Not serious	Not serious	Very serious‡	None	0/101 (0.0%)	0/105 (0.0%)	Not estimable. No deaths due to cardiovascular disease occurred	⨁◯◯◯ Very low*‡
Hospitalisation due to heart failure										
1	Randomised trials	Serious*	Not serious	Not serious	Very serious†	None	1/19 (5.3%)	0/20 (0.0%)	One study reported a single hospitalisation for heart failure, in a person receiving CoQ10	⨁◯◯◯ Very low*†
Quality of life (QOL, assessed with: Kansas City Cardiomyopathy Questionnaire; scale from: 0 to 100, lower scores represent worse symptoms. Minimally important difference considered to be 5 points^37^										
2	Randomised trials	Serious*	Serious§	Not serious	Serious¶	None	68	71	One study showed QOL was −8.52 points lower (95% CI −19.20 to 2.16**) and the other showed QOL was 22.41 points higher (95% CI 12.18 to 32.64) for those taking CoQ10	⨁◯◯◯ Very low*§¶

* Downgraded by one level due to risk of bias.

† Downgraded two levels as number of participants and events are extremely low, consistent with the possibility of both benefit and harm from the intervention.

‡ No effects in either group, so estimate of effects cannot be calculated, and number of participants is extremely low.

§ Downgraded by one level as direction of effect varied. One study reported significant improvement, and one reported a trivial change in QOL.

¶ Meta-analysis not conducted due to clinical heterogeneity (one study compared CoQ10 to placebo, one used D-ribose as a co-intervention in both groups, which may account for the variation in results).

** Note that the variance for this contrast was estimated for this contrast.

CoQ10, coenzyme Q10; KCCQ, Kansas City Cardiomyopathy Questionnaire.

### Primary outcomes

All included trials reported mortality, including cardiovascular mortality. Only two deaths were reported, and neither was due to cardiovascular disease (one was due to sepsis,^32^ the other was described as an ‘unrelated’ death^34^). Both of these occurred in groups receiving CoQ10 (mortality 2/101 participants receiving CoQ10 vs 0/105 participants receiving placebo). Given the scarcity of data, we did not conduct any meta-analysis. The small numbers of participants, extremely low event rate and concerns over the risk of bias in the included studies mean that this evidence was considered very low certainty.

Only one study reported hospitalisation due to HF. Samuel *et al*^32^ reported a single hospitalisation due to HF in a participant receiving CoQ10 (1/19 participants receiving CoQ10 vs 0/20 participants receiving placebo). The certainty of evidence was considered very low due to the risk of bias and imprecision.

Quality of life was reported by a single study,^34^ using the KCCQ. Scores range from 0 to 100, and higher scores represent improvement, with a change of 5 points considered meaningful.^37^ Comparison of CoQ10 versus placebo in this study suggested a possible improvement (mean difference (MD) 22.41; 95% CI 12.18 to 32.64, 73 participants), whereas CoQ10+D-ribose versus D-ribose alone showed no additional benefit (MD −8.52; 95% CI −19.20 to 2.16, 66 participants, variance for this contrast was imputed) (figure 2). The certainty of the evidence was judged very low, due to the risk of bias, inconsistency in results and imprecision in the effect estimates.

**Figure 2:**
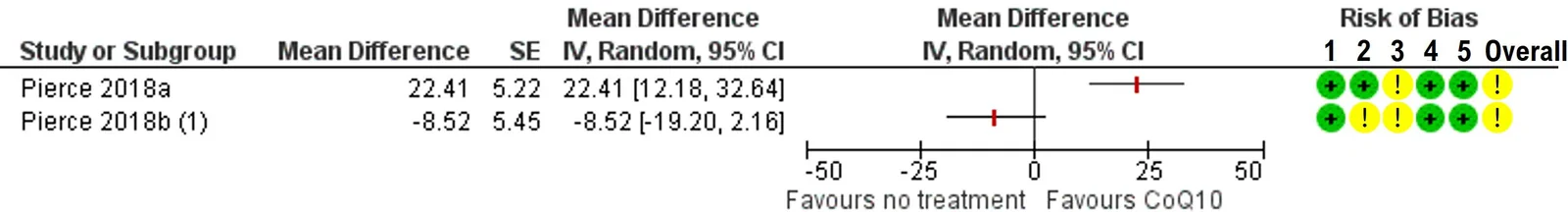
This study included D-ribose as a cointervention for both groups. Coloured circles denote assessed risk of bias for each domain (1–5) and overall: green indicates low risk of bias, yellow indicates some concerns regarding risk of bias. Risk of bias assessment considered five domains: 1—bias arising from the randomisation process; 2—bias due to deviations from intended interventions; 3—bias due to missing outcome data; 4—bias in the measurement of the outcome; 5—bias in the selection of the reported result. The final column represents the overall risk of bias for the study result. (1) This comparison represents CoQ10 plus D-ribose versus D-ribose alone. CoQ10, coenzyme Q10.

### Secondary outcomes

Samuel *et al*^32^ reported two hospitalisations in the CoQ10 group (out of 19 participants; one non-ST elevation myocardial infarction, one exacerbation of HF) compared with one hospitalisation in the control group (out of 20 participants; non-ST elevation acute coronary syndrome) after 4 months of follow-up.

In Pierce *et al*,^34^ the 6-minute walk test showed no meaningful difference between those receiving CoQ10 and placebo (mean difference 1.59 m, 95% CI −27.71 to 30.89, 73 participants) or between those receiving CoQ10 plus D-ribose versus D-ribose alone (MD 6.79 m, 95% CI −21.69 to 35.27, 66 participants, variance for this contrast was imputed). The minimally important difference for the 6-minute walk test has been suggested as between 14 and 30 m for adults with chronic disease.^38^

Two studies^32 34^ reported changes in biomarkers. Pierce *et al*^29^ reported a change in BNP level. Those receiving CoQ10 had a reduction in BNP level compared with those receiving placebo (MD −72.02 pg/mL, 95% CI −114.8 to −29.21, 73 participants). No significant difference was seen when comparing those who received CoQ10 plus D-ribose to those receiving D-ribose alone (MD 11.13, 95% CI −24.25 to 46.51, 66 participants, variance for this contrast was imputed). Again, given the scarcity of data and differing directions of effect we do not provide a pooled estimate of these data.

Samuel *et al*^32^ reported changes in NTproBNP levels. There was no significant reduction in NTproBNP for participants receiving CoQ10 (MD −223 pg/mL, 95% CI −1093.81 to 647.81, 39 participants).

NYHA class was not reported by any of the included studies.

Adverse events were reported by all three studies. Comparing CoQ10 to placebo resulted in a risk ratio of 1.44 (95% CI 0.79 to 2.62, I^2^=0%, 208 participants, adverse events in 21/102 participants receiving CoQ10, 15/106 receiving placebo) (figure 3). Adverse events include cardiac disorders, gastrointestinal disorders, infections and infestations, metabolism and nutritional disorders, musculoskeletal and connective tissue disorders, nervous system disorders, renal and urinary disorders, and respiratory, thoracic and mediastinal disorders.

**Figure 3:**
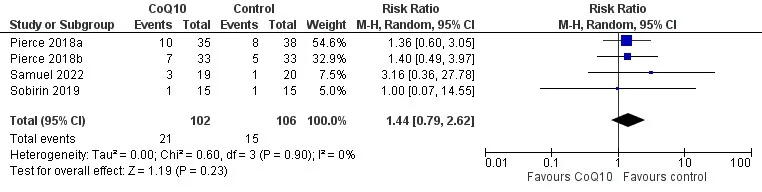
Pierce 2018a and 2018b^36^ included D-ribose as a cointervention for both groups. This outcome was secondary, thus, risk of bias was not conducted for this outcome. CoQ10, coenzyme Q10; M-H, Mantel-Haenszel.

As such limited data were identified, we were unable to carry out our planned subgroup analyses according to age, sex and comorbidities.

## Discussion

### Interpretation of the results

This review is the first systematic review and meta-analysis to focus exclusively on randomised controlled trials evaluating CoQ10 supplementation in adults with HFpEF. Previous reviews have primarily included HFrEF or mixed HF populations. Synthesising evidence specific to HFpEF is essential to inform clinical practice and future research. By restricting inclusion to HFpEF populations, this review addresses an important gap in existing literature.

Despite biological plausibility for CoQ10 as a therapy targeting impaired myocardial energetics, the current evidence base in HFpEF is extremely limited. Only three small randomised controlled trials, including 222 participants in total, met inclusion criteria. Across all outcomes, sample sizes were small, event rates were rare and the overall certainty of evidence was judged very low. At present, the data are uninformative regarding whether CoQ10 supplementation is a useful adjunct for those with HFpEF.

Mortality was infrequent, with only two deaths reported across studies. Both occurred in participants receiving CoQ10, but neither was attributable to cardiovascular causes. Hospitalisation for HF was reported for a single participant (also receiving CoQ10) in one study. Similarly, only one study assessed the effect of CoQ10 on quality of life, and results were inconsistent (between those who did and did not receive D-ribose as a cointervention). The low number of hospitalisations and deaths is not surprising given the short follow-up across trials (maximum 4 months) and the fact that most participants had NYHA class II–III. However, collectively, these results emphasise the lack of adequately powered studies assessing important clinical endpoints in HFpEF populations.

Select trials in HFrEF, notably Q-SYMBIO,^21^ have shown that CoQ10 improved symptoms and clinical outcomes in people with HFrEF. These studies support the biological plausibility of benefit through improved bioenergetics and CoQ10’s antioxidant activity. A recent systematic review and cost-effectiveness study, including 26 trials, on CoQ10 in HFrEF populations concluded that CoQ10 is cost-effective in HFrEF.^22^ Specifically, CoQ10 was found to have an incremental cost-effectiveness ratio of £3650 per quality-adjusted life year (QALY). A cost of £20 000–£30 000 per QALY is acceptable according to the National Institute for Health and Care Excellence guidance.^39^ An ongoing NIHR-funded RCT, the CORAL study (NIHR152257),^40^ is currently evaluating clinical outcomes of CoQ10 supplementation in HFrEF.

Still, HFpEF remains understudied, not only for CoQ10 but for pharmacological therapies in general. HFrEF trials benefit from a more well-defined, homogenous condition, with clear mechanistic targets and endpoints. HFpEF is heterogeneous, mechanistically complex, with less established evidence of disease-modifying therapies and fewer mechanistic targets, reflecting the inherent challenges of HFpEF research.

### Limitations of the evidence

Available trials were small and short in duration, heterogeneous, with some concerns regarding the risk of bias and inconsistent outcome reporting. They also varied in CoQ10 formulations, doses and co-supplementations. Event rates were extremely low, and important outcomes—including NYHA class and longer-term endpoints—were not reported at all. Additionally, the inclusion of trials combining CoQ10 with D-ribose introduces potential effect modification, further complicating interpretation as these both act on ATP production through different pathways. Collectively, these limitations constrain both validity and generalisability, and result in very low certainty of evidence for CoQ10 in HFpEF.

The available evidence in this area comes from three small studies. It is possible that additional trials have been conducted but remain unpublished due to their findings (publication bias). In principle, the small size of these studies also gives the potential risk of small study effects—where smaller studies in a meta-analysis show larger effects.^29^ Conducting larger, methodologically rigorous trials that recruit participants with more diverse clinical features may avoid this concern in future.

There are many reasons for the limited evidence base of CoQ10 for HFpEF. It was only relatively recently that HFpEF was recognised as a distinct clinical phenotype of HF, and as a result, very few CoQ10 trials have been designed specifically for HFpEF. The heterogeneity of the HFpEF population (typically older people with a wider variety of comorbidities) might make it harder for a single intervention like CoQ10 to show clear benefit, especially on hard endpoints such as cardiovascular events and mortality.

### Limitations of the review process

This review followed a prospectively registered protocol and applied comprehensive multi-database searches, translation of non-English records, duplicate screening and data extraction. Despite this, certain procedural constraints remain. Some unpublished or inaccessible trials could not be retrieved despite contacting the authors, introducing potential publication bias. Reliance on machine translation (DeepL and Google Translate) over human translation could also introduce minor inaccuracies. We chose not to conduct meta-analysis for our primary outcomes. Given the extreme scarcity of events, including mortality and hospitalisation for HF, we considered that any calculations of pooled effect estimates and their variance may be unreliable. Challenges with meta-analysis of single-zero and double-zero trials are well known, and resulting effect estimates and CIs may be highly sensitive to the analysis model chosen.^41^ It is clear from the raw data that there is great uncertainty in the effectiveness of CoQ10, and we considered that a meta-analysis (resulting in a potentially biased effect estimate, with very large CIs) would not aid decision-making in this area. Nonetheless, others may have taken a different approach and provided a statistical summary.

### Implications

At present, evidence regarding the use of CoQ10 for individuals with HFpEF is very uncertain. Few trials have focused specifically on this population, and the available evidence is sparse. Future trials need to be multicentre to improve generalisability. There should also be a standardised CoQ10 formulation, dosing and duration.

Given the growing prevalence of HFpEF and the lack of effective therapies, further investigation into CoQ10 remains justified.

## Conclusions

Evidence from existing RCTs does not allow a definitive conclusion on the effectiveness and safety of CoQ10 in HFpEF. These studies are limited in sample size, short in duration and there is considerable heterogeneity in both design and outcome measures. Despite these limitations, the biological rationale for the therapeutic effect of CoQ10 remains strong, due to its core role in mitochondrial energy metabolism and its beneficial effects reported in HF reviews encompassing all EFs. Given the mechanistic overlap between HFrEF and HFpEF, and the supporting evidence observed in HFrEF, CoQ10 supplementation seems a biologically plausible option in HFpEF. This review, therefore, highlights a gap in the existing evidence. To clarify the therapeutic potential of CoQ10 in HFpEF, future trials must use larger sample sizes, have multicentre designs and rigorous methodologies.

## Supplementary material

10.1136/bmjopen-2025-116033 online supplemental file 1

## Data Availability

All data relevant to the study are included in the article or uploaded as supplementary information.
