# Supplementary material for: Effectiveness and safety of coenzyme Q10 in people with heart failure with preserved ejection fraction (HFpEF): a systematic review and meta-analysis
Source: BMJ Open. 2026 Oct 13;16(10):e116033. doi: 10.1136/bmjopen-2025-116033 (PMC13663742; doi:10.1136/bmjopen-2025-116033)
Supplement: Supplementary file 1 [file bmjopen-2025-116033.reviewer_comments.pdf]

PEER REVIEW HISTORY

BMJ Open publishes all reviews undertaken for accepted manuscripts. Reviewers are asked to complete a checklist review form and are provided with free text boxes to elaborate on their assessment. These free text comments are reproduced below.

ARTICLE DETAILS

Title (Provisional)

The Effectiveness and Safety of Coenzyme Q10 in People with Heart Failure with Preserved Ejection Fraction (HFpEF): A Systematic Review and Meta-Analysis

Authors

Rahman, Ebadur; Webster, Katie E; Nightingale, Angus K; Pufulete, Maria

---

VERSION 1 - REVIEW

**Reviewer**	**1**
**Name**	**Noma, Hisashi**
**Affiliation**	**The Institute of Statistical Mathematics, Department of Interdisciplinary Statistical Mathematics**
**Date**	**24-Feb-2026**
**COI**	**None**

---

1. The primary statistical concern relates to the handling of extremely sparse event data. Across the included trials, the total number of deaths is two, with zero events in the control group for some outcomes. Under such conditions, conventional relative risk estimation is known to be unstable and highly sensitive to the choice of correction. In particular, when one or more studies contain zero events in one arm, standard Haldane-type corrections (e.g., adding 0.5 to each cell) can substantially distort both point estimates and confidence intervals. The resulting relative risk estimates may therefore reflect the correction method more than the underlying data. Given that the total information size is extremely small, the reported pooled estimates (e.g., RR 3.07 for all-cause mortality) should be interpreted with extreme caution. To address this issue, I strongly recommend re-analysing the rare-event outcomes using an exact hypergeometric-normal model as implemented in the R package “metafor.” This approach models the within-study likelihood using the hypergeometric distribution (i.e., conditional on the total number of events), thereby avoiding arbitrary continuity corrections and providing more appropriate inference in sparse binary data settings.

2. The manuscript reports I^2 statistics (e.g., I^2 = 0% for mortality). While technically correct, I would caution against interpreting I^2 as evidence of homogeneity in this context. When

event rates are extremely low and the number of studies is very small (k = 2–3), I^2 has very limited interpretability. In such situations, the estimate of between-study variance is driven by minimal information, and I^2 may default to 0% simply due to insufficient statistical power to detect heterogeneity. I recommend that the authors explicitly acknowledge in the Results or Discussion that I^2 is not informative under conditions of sparse events and small k, and that "I^2 = 0%" should not be interpreted as evidence of true homogeneity.

3. The manuscript refrains from pooling KCCQ results due to differences in direction of effect and clinical heterogeneity (use of D-ribose in one comparison). The decision is reasonable, but the justification could be strengthened statistically. Formally, a random-effects model can accommodate variation in direction and magnitude of effect. Therefore, the decision not to pool should be framed explicitly as a judgment based on clinical heterogeneity (co-intervention with D-ribose) rather than statistical impossibility. Clarifying that the choice was based on concerns regarding effect modification would improve methodological transparency. The authors may also consider presenting the two effect estimates side-by-side with a brief explanation that pooling was avoided due to anticipated interaction between CoQ10 and D-ribose.

4. For the Pierce 2018 factorial trial, the variance of an unreported contrast was approximated by averaging the variances of other reported contrasts. While pragmatic, this approach introduces strong assumptions:

- It assumes homoscedasticity across comparisons.
- It ignores potential covariance between factorial arms.
- It may underestimate uncertainty if variance differs across contrasts.

Given the small number of trials overall, even modest underestimation of variance could materially influence pooled estimates.

I recommend that the authors conduct a sensitivity analysis in which:

- The approximated variance is inflated (e.g., multiplied by 1.5 or 2.0), or
- The comparison requiring imputed variance is excluded.

This would allow readers to assess the robustness of the findings to the variance approximation strategy.

5. In the Summary of Findings table, absolute effects appear to be calculated using an assumed baseline risk (e.g., 5%). The rationale for selecting this baseline risk should be explicitly justified. Given that observed event rates were extremely low, the assumed baseline risk materially affects the magnitude of absolute risk differences. If possible, the authors should clarify whether the baseline risk was derived from control-arm data or chosen for illustrative purposes. Transparent reporting of this choice is important, particularly when event numbers are minimal.

6. Although funnel plot–based assessments are not feasible with only three trials, it would be appropriate to explicitly state that small-study effects and publication bias could not be formally evaluated due to the limited number of included studies.

Given the small sample sizes and short follow-up durations of the included trials, the potential for unpublished null studies should be acknowledged.

7. For mortality and hospitalization outcomes, point estimates of the relative risk exceed 1.0. While the authors correctly emphasize the very low certainty of evidence, care should be taken to avoid language that could be interpreted as suggesting possible harm.

Given the extreme imprecision and near-absence of events, it may be preferable to state that the data are "uninformative" rather than implying directional trends.

**Reviewer**	**2**
**Name**	**Badrin, Salziyan**
**Affiliation**	**Universiti Sains Malaysia - Kampus Kesihatan, Department of Family Medicine**
**Date**	**26-Mar-2026**
**COI**	**None**

Thank you for giving me the opportunity to review this manuscript. Here are my comments:

1. In the 'Methods' section (page 5), the authors did not mention language restrictions as inclusion or exclusion criteria. In the PRISMA flow diagram (page 23), the authors presented a diagram of 'identification of study from other methods' but did not specifically describe it in the 'Methods' section in the manuscript. What 'other methods' did the authors use to identify the studies for inclusion?

2. In Page 7, line 23, the author mentioned the I statistic but did not clearly write how the authors determined the percentages of heterogeneity?

3. In Table 2, the authors did not include a legend explaining the colour coding used in the table.

4. There is a spelling error in the line 47, page 13: "Pierce (2018) reported the chfiguange in BNP level".

**Reviewer**	**3**
**Name**	**Tchana, Bertrand**
**Affiliation**	**Azienda Ospedaliero-Universitaria di Parma, Bertrand Tchana**
**Date**	**03-May-2026**

COI **None**

This study is a well-conducted review addressing a clinically relevant question: Ubiquinone in heart failure with preserved ejection fraction. It needs some clarification, the risk of bias assessment, refinement in the interpretation and conclusions.

VERSION 1 - AUTHOR RESPONSE

Reviewer: 1
Prof. Hisashi Noma, The Institute of Statistical Mathematics, The Graduate University for Advanced Studies

Comments to the Author:
1. The primary statistical concern relates to the handling of extremely sparse event data. Across the included trials, the total number of deaths is two, with zero events in the control group for some outcomes. Under such conditions, conventional relative risk estimation is known to be unstable and highly sensitive to the choice of correction. In particular, when one or more studies contain zero events in one arm, standard Haldane-type corrections (e.g., adding 0.5 to each cell) can substantially distort both point estimates and confidence intervals. The resulting relative risk estimates may therefore reflect the correction method more than the underlying data. Given that the total information size is extremely small, the reported pooled estimates (e.g., RR 3.07 for all-cause mortality) should be interpreted with extreme caution. To address this issue, I strongly recommend re-analysing the rare-event outcomes using an exact hypergeometric-normal model as implemented in the R package "metafor." This approach models the within-study likelihood using the hypergeometric distribution (i.e., conditional on the total number of events), thereby avoiding arbitrary continuity corrections and providing more appropriate inference in sparse binary data settings.

Thanks for this important comment. We agree that the sparse data, with few studies and very rare events makes reliable statistical analysis challenging. We have looked carefully at alternative analysis options. The data are even too sparse for the hypergeometric-normal model in the metafor package, as recommended by the reviewer. We would like to thank our colleague, Professor Julian Higgins, for a helpful discussion on the meta-analysis of sparse data.

After discussion and reflection, we agree that meta-analysis with such scarce data (2-3 studies, only one or zero events per study arm) is likely to be uninformative and unhelpful, regardless of the statistical approach taken. Instead, we have opted to present a narrative summary of the findings for the analyses with very sparse data (mortality, hospitalisation for heart failure and any hospitalisation). Highlighting the uncertainty in the results. We hope that this is an acceptable approach.

2. The manuscript reports I^2 statistics (e.g., I^2 = 0% for mortality). While technically correct, I would caution against interpreting I^2 as evidence of homogeneity in this context. When event rates are extremely low and the number of studies is very small (k = 2–3), I^2 has very limited interpretability. In such situations, the estimate of between-study variance is driven by minimal information, and I^2 may default to 0% simply due to insufficient statistical power to detect heterogeneity. I recommend that the authors explicitly acknowledge in the Results or Discussion that I^2 is not informative under conditions of sparse events and small k, and that "I^2 = 0%" should not be interpreted as evidence of true homogeneity.

Thanks for the comment. As many of the meta-analyses have now been replaced with a narrative synthesis/synthesis without meta-analysis, this issue has largely been resolved. We agree that the small number of studies in the previous analyses meant that I^2 could not be used to infer homogeneity between the trials and apologise if this was the impression given.

3. The manuscript refrains from pooling KCCQ results due to differences in direction of effect and clinical heterogeneity (use of D-ribose in one comparison). The decision is reasonable, but the justification could be strengthened statistically. Formally, a random-effects model can accommodate variation in direction and magnitude of effect. Therefore, the decision not to pool should be framed explicitly as a judgment based on clinical heterogeneity (co-intervention with D-ribose) rather than statistical impossibility. Clarifying that the choice was based on concerns regarding effect modification would improve methodological transparency. The authors may also consider presenting the two effect estimates side-by-side with a brief explanation that pooling was avoided due to anticipated interaction between CoQ10 and D-ribose.

Thank you for encouraging us to clarify this. Although both CoQ10 and D-ribose target myocardial energetics, they act through different mechanisms: CoQ10 supports mitochondrial oxidative phosphorylation, whereas D-ribose provides substrate for adenine nucleotide synthesis and ATP replenishment. As the RCT by Pearce et al evaluated CoQ10 in combination with D-ribose rather than CoQ10 alone, we considered the co-intervention could plausibly modify the treatment effect, making the estimates clinically non-comparable. We have therefore followed the reviewer's suggestion to present the two effect estimates side-by-side and have clarified in the manuscript that pooling was avoided because of anticipated clinical heterogeneity and the potential for effect modification, rather than because random-effects meta-analysis could not be performed.

4. For the Pierce 2018 factorial trial, the variance of an unreported contrast was approximated by averaging the variances of other reported contrasts. While pragmatic, this approach introduces strong assumptions:

• It assumes homoscedasticity across comparisons.
• It ignores potential covariance between factorial arms.
• It may underestimate uncertainty if variance differs across contrasts.
Given the small number of trials overall, even modest underestimation of variance could materially influence pooled estimates.
I recommend that the authors conduct a sensitivity analysis in which:
• The approximated variance is inflated (e.g., multiplied by 1.5 or 2.0), or
• The comparison requiring imputed variance is excluded.
This would allow readers to assess the robustness of the findings to the variance approximation strategy.

Thank you for this suggestion. As we have now presented individual effect estimates from these two contrasts, the estimated variance does not impact on any pooled estimates of effect (therefore we are unable to conduct any formal sensitivity analyses with inflated variance or excluding the imputed data). Instead we have highlighted this issue in the methods section, and also identified the relevant effect estimates in the results. We hope that this is sufficient to convey the uncertainty in the data.

5. In the Summary of Findings table, absolute effects appear to be calculated using an assumed baseline risk (e.g., 5%). The rationale for selecting this baseline risk should be explicitly justified. Given that observed event rates were extremely low, the assumed baseline risk materially affects the magnitude of absolute risk differences. If possible, the authors should clarify whether the baseline risk was derived from control-arm data or chosen for illustrative purposes. Transparent reporting of this choice is important, particularly when event numbers are minimal.

The baseline risk of 5% was chosen for illustrative purposes. However, as the analysis of these outcomes have now been revised to provide a narrative summary of the data, we present only the actual, raw event rates and total number of participants in the summary of findings table. We hope that this provides a more accurate representation of the data.

6. Although funnel plot–based assessments are not feasible with only three trials, it would be appropriate to explicitly state that small-study effects and publication bias could not be formally evaluated due to the limited number of included studies. Given the small sample sizes and short follow-up durations of the included trials, the potential for unpublished null studies should be acknowledged.

Thank you – we have now added some text to the section in the discussion on ‘limitations of the evidence’ to address these points.

7. For mortality and hospitalization outcomes, point estimates of the relative risk exceed 1.0. While the authors correctly emphasize the very low certainty of evidence, care should be taken to avoid language that could be interpreted as suggesting possible harm.
Given the extreme imprecision and near-absence of events, it may be preferable to state that the data are "uninformative" rather than implying directional trends.

Thank you. We hope that the updated analysis and consequent revisions to the language have alleviated this concern. We have also added a specific sentence to the discussion to highlight this: "At present, the data are uninformative regarding whether CoQ10 supplementation is a useful adjunct for those with HFpEF."

Reviewer: 2
Dr. Salziyan Badrin, Universiti Sains Malaysia - Kampus Kesihatan
Comments to the Author:
Thank you for giving me the opportunity to review this manuscript. Here are my comments:

1. In the 'Methods' section (page 5), the authors did not mention language restrictions as inclusion or exclusion criteria. In the PRISMA flow diagram (page 23), the authors presented a diagram of 'identification of study from other methods' but did not specifically describe it in the 'Methods' section in the manuscript. What 'other methods' did the authors use to identify the studies for inclusion?

Thanks for the opportunity to clarify this. Given the existence of a recent Cochrane review in a related (but much broader) topic area, we searched for articles using two methods. Firstly, we searched the reference lists of the Cochrane review (both included and excluded studies) to identify articles that should be assessed at full text. This is the step referred to in the PRISMA diagram as 'identification of studies via other methods'. Then we ran an updated search to capture additional articles published since the original searches in the Cochrane review (referred to as 'identification of studies via databases and registers'). We have added some additional text to the start of the results section which hopefully clarifies this for the reader: "The full text of 71 articles was assessed*, including 34 records identified from an existing Cochrane review (of CoQ10 for all types of heart failure), and an additional 37 records retrieved by our new searches.*"

2. In Page 7, line 23, the author mentioned the I statistic but did not clearly write how the authors determined the percentages of heterogeneity?

Thanks, we have now added references to indicate how the I^2 statistic was calculated. However, please note the response to the reviewer above, where we highlight that we now only conduct a meta-analysis for adverse event data, and present narrative syntheses for other outcomes.

3. In Table 2, the authors did not include a legend explaining the colour coding used in the table.
Apologies for this omission – we have now added a description of the colour coding.

4. There is a spelling error in the line 47, page 13: "Pierce (2018) reported the chfiguange in BNP level".

Thank you for spotting this error. It has now been corrected.

Reviewer: 3
Dr. Bertrand Tchana, Azienda Ospedaliero-Universitaria di Parma
Comments to the Author:
This study is a well-conducted review addressing a clinically relevant question: Ubiquinone in heart failure with preserved ejection fraction. It needs some clarification, the risk of bias assessment, refinement in the interpretation and conclusions.

Thanks for the appreciation of the review. We believe that the edits made in response to the reviewers' helpful comments have markedly improved the manuscript.

VERSION 2 - REVIEW

**Reviewer**	**1**
**Name**	**Noma, Hisashi**
**Affiliation**	**The Institute of Statistical Mathematics, Department of Interdisciplinary Statistical Mathematics**
**Date**	**01-Aug-2026**
**COI**	

Thank you for your careful and thorough revisions; the authors have satisfactorily addressed my comments, and I have no further concerns.

VERSION 2 - AUTHOR RESPONSE

Reviewer: 1
Prof. Hisashi Noma, The Institute of Statistical Mathematics, The Graduate University for Advanced Studies
Comments to the Author:
Thank you for your careful and thorough revisions; the authors have satisfactorily addressed my comments, and I have no further concerns.

Thanks for this positive review, and for your helpful comments on the original submission.

Reviewer: 1
If you have selected 'Yes' above, please provide details of any competing interests.: Not applicable.
