# Supplementary material for: Effectiveness and safety of coenzyme Q10 in people with heart failure with preserved ejection fraction (HFpEF): a systematic review and meta-analysis
Source: BMJ Open. 2026 Oct 13;16(10):e116033. doi: 10.1136/bmjopen-2025-116033 (PMC13663742; doi:10.1136/bmjopen-2025-116033)
Supplement: Supplementary file 2 [file bmjopen-16-10-s001.docx]

**Search Strategies**

**CENTRAL:** date restricted from 16/10/2020

#1 MeSH descriptor: [Ubiquinone] this term only

#2 ubiquinon*

#3 ubidecarenone

#4 q10 or "q 10"

#5 coq10 or "coq 10"

#6 coenzyme next Q*

#7 co-enzyme next Q*

#8 neuquinon*

#9" quinone"

#10 ubiquinol

#11 Bio-Quinone next Q10

#12 (#1 or #2 or #3 or #4 or #5 or #6 or #7 or #8 or #9 or #10 or #11)

#13 MeSH descriptor: [Heart Failure] explode all trees

#14 heart next failure*

#15 cardiac next failure*

#16 cardiomyopath*

#17 #13 or #14 or #15 or #16

#18 #12 and #17

**ISI Web of Science:** date restricted from 16/10/2020

#1 TS=(q10 or coq10)

#2 TS=("q 10" or "coq 10")

#3 TS=ubiquinon*

#4 TS=ubidecarenone

#5 TS=("coenzyme Q*" or "co-enzyme Q*")

#6 TS=neuquinon*

#7 TS=quinone

#8 TS=ubiquinol

#9 TS="Bio-Quinone Q10"

#10 #9 OR #8 OR #7 OR #6 OR #5 OR #4 OR #3 OR #2 OR #1

#11 TS=(heart SAME failure*)

#12 TS=(cardiac SAME failure*)

#13 TS=(myocardial SAME failure*)

#14 TS=cardiomyopath*

#15 #14 OR #13 OR #12 OR #11

#16 #15 AND #10 Timespan 2020-2025

**CINAHL:** date restricted from 16/10/2020

S40 S21 and S39 Published Date: 20201031-20250625

S39 S22 or S23 or S24 or S25 or S26 or S27 or S28 or S29 or S30 or S31 or S32 or S33 or S34 or S35 or S36 or S37 or S38

S38 TX cross-over*

S37 TX crossover*

S36 TX volunteer*

S35 (MH "Crossover Design")

S34 TX allocat*

S33 TX control*

S32 TX assign*

S31 TX placebo*

S30 (MH "Placebos")

S29 TX random*

S28 TX (doubl* N1 mask*)

S27 TX (singl* N1 mask*)

S26 TX (doubl* N1 blind*)

S25 TX (singl* N1 blind*)

S24 TX (clinic* N1 trial?)

S23 PT clinical trial

S22 (MH "Clinical Trials+")

S21 S14 and S20

S20 S15 or S16 or S17 or S18 or S19

S19 (TI cardiomyopath*) or (AB cardiomyopath*)

S18 (TI (myocardial N2 failure*)) or (AB (myocardial N2 failure*))

S17 (TI (cardiac N2 failure*)) or (AB (cardiac N2 failure*))

S16 (TI (heart N2 failure*)) or (AB (heart N2 failure*))

S15 (MH "Heart Failure +")

S14 S1 or S2 or S3 or S4 or S5 or S6 or S7 or S8 or S9 or S10 or S11 or S12 or S13 S13 (TI coq 10) or (AB coq 10)

S12 (TI q 10) or (AB q 10) S11 (TI Bio-Quinone Q10) or (AB Bio-Quinone Q10) S10 (TI ubiquinol) or (AB ubiquinol)

S9 (TI quinone) or (AB quinone)

S8 (TI neuquinon*) or (AB neuquinon*)

S7 (TI co-enzyme Q*) or (AB co-enzyme Q*)

S6 (TI coenzyme Q*) or (AB coenzyme Q*)

S5 (TI coq10) or (AB coq10)

S4 (TI ubidecarenone) or (AB ubidecarenone)

S3 (TI ubiquinon*) or (AB ubiquinon*)

S2 (TI q10) or (AB q10)

S1 (MH "Coenzyme Q")

**AMED:** date restricted from 2020-2025

1. coenzymes/
2. Ubiquinone.tw.
3. ubidecarenone.tw.
4. Q10.tw.
5. CoQ10.tw.
6. coenzym$ Q$.tw.
7. co-enzym$ Q$.tw.
8. neuquinon$.tw.
9. quinone.tw.
10. ubiquinol.tw.
11. Q-10.tw.
12. or/1-11
13. heart failure congestive/
14. heart failure.tw.
15. cardiac failure.tw.
16. cardiomyopathies/
17. cardiomyopath$.tw.
18. or/13-17
19. 12 and 18
20. limit 19 to yr="2020-2025"

**MEDLINE Ovid:** date restricted from 16/10/2020

1. Ubiquinone/
2. ubiquinon*.tw.
3. ubidecarenone.tw.
4. q10.tw.
5. coq10.tw.
6. coenzyme Q*.tw.
7. co-enzyme Q*.tw.
8. neuquinon*.tw.
9. quinone.tw.
10. ubiquinol.tw.
11. Bio-Quinone Q10.tw.
12. q 10.tw.
13. coq 10.tw.
14. or/1-13
15. exp Heart Failure/
16. (heart adj2 failure*).tw.
17. (cardiac adj2 failure*).tw.
18. (myocardial adj2 failure*).tw.
19. cardiomyopath*.tw.
20. or/15-19
21. 14 and 20
22. randomized controlled trial.pt.
23. controlled clinical trial.pt.
24. randomized.ab.
25. placebo.ab.
26. drug therapy.fs.
27. randomly.ab.
28. trial.ab.
29. groups.ab.
30. 22 or 23 or 24 or 25 or 26 or 27 or 28 or 29
31. exp animals/ not humans.sh.
32. 30 not 31
33. 21 and 32
34. limit 33 to ed=20201016-20250625
35. ((preserve* or normal or greater) adj5 (ejection fraction or EF or LVEF)).mp.
36. (HFpEF or HF-pEF).tw,kf.
37. 35 or 36
38. 34 and 37
39. 34 or 38

**EMBASE Ovid:** date restricted from 16/10/2020

1. ubidecarenone/
2. ubiquinone/
3. ubiquinon*.tw.
4. ubidecarenone.tw.
5. q10.tw.
6. coq10.tw.
7. coenzyme Q*.tw.
8. co-enzyme Q*.tw.
9. neuquinon*.tw.
10. quinone.tw.
11. ubiquinol.tw.
12. Bio-Quinone Q10.tw.
13. q 10.tw.
14. coq 10.tw.
15. or/1-14
16. exp heart failure/
17. (heart adj2 failure*).tw.
18. (cardiac adj2 failure*).tw.
19. (myocardial adj2 failure*).tw.
20. cardiomyopath*.tw.
21. or/16-20
22. 15 and 21
23. random$.tw.
24. factorial$.tw.
25. crossover$.tw.
26. cross over$.tw.
27. cross-over$.tw.
28. placebo$.tw.
29. (doubl$ adj blind$).tw.
30. (singl$ adj blind$).tw.
31. assign$.tw.
32. allocat$.tw.
33. volunteer$.tw.
34. crossover procedure/
35. double blind procedure/
36. randomized controlled trial/
37. single blind procedure/
38. or/23-37
39. (animal/ or nonhuman/) not human/
40. 38 not 39
41. 22 and 40
42. limit 41 to dd=20201016-20250613
43. ((preserve* or normal or greater) adj5 (ejection fraction or EF or LVEF)).mp.
44. (HFpEF or HF-pEF).tw,kf.
45. 43 or 44
46. 42 and 45
47. 42 or 46
48. 42 or 46
49. limit 41 to dc=20201016-20250625
50. 202*.yr,dd,dc.
51. 41 and 50
52. 51 not 49
53. 42 or 49

**ISRCTN:**

("coenzyme Q" OR "Ubiquinone" OR "ubidecarenone" OR "Q10" OR "CoQ10" OR "coenzyme Q" OR "co-enzyme" OR "Q neuquinon" OR "quinone" OR "ubiquinol Q-10") AND ("heart failure" OR "congestive heart failure" OR "cardiac failure" OR "cardiomyopathies" OR "cardiomyopath")

**NIH (ClinicalTrials.gov):** date restricted from 01/16/2020

"heart failure" OR "congestive heart failure" OR "cardiac failure" OR "cardiomyopathies" OR "cardiomyopath" | Other terms: "coenzyme Q" OR "Ubiquinone" OR "ubidecarenone" OR "Q10" OR "CoQ10" OR "coenzyme Q" OR "co-enzyme" OR "Q neuquinon" OR "quinone" OR "ubiquinol Q-10"

**Excluded studies**

1. NCT06694727. A Danish Pragmatic Randomized Trial of Nutritional Supplements in Heart Failure (DANUTRIO-HF). <https://clinicaltrials.gov/study/NCT06694727> (first posted 19 November 2024)
2. NCT01925937. Atorvastatin/ CoenzymeQ10 in Congestive Heart Failure. <https://clinicaltrials.gov/study/NCT01925937> (first posted 20 August 2013)
3. ISRCTN20190612043878. Effect of Coenzyme Q10 on echocardiographic parameters. <https://trialsearch.who.int/Trial2.aspx?TrialID=IRCT20190612043878N1> (date assigned 14 April 2014)
4. Urban Alehagen, Johansson P, Svensson E, Aaseth J, Alexander J. Improved cardiovascular health by supplementation with selenium and coenzyme Q10: applying structural equation modelling (SEM) to clinical outcomes and biomarkers to explore underlying mechanisms in a prospective randomized double-blind placebo-controlled intervention project in Sweden. European journal of nutrition. 2022 Apr 6;61(6):3135–48
5. Bodea O, Eugen Radu Boia, Craciun LM, Mihaela Daniela Valcovici, Alexandru Catalin Motofelea, Munteanu AM, et al. Effect of Coenzyme Q10 Supplementation on Cardiac Function and Quality of Life in Patients with Heart Failure: A Randomized Controlled Trial. Journal of Clinical Medicine. 2025 May 23;14(11):3675–5.
6. Keogh A, Fenton S, Leslie C, Aboyoun C, Macdonald P, Yi Chen Zhao, et al. Randomised double-blind, placebo-controlled trial of coenzyme Q10 therapy in class II and III systolic heart failure. Heart, Lung and Circulation. 2003 Jan;12(3):135–41.
7. Pourrmoghaddas M, Rabbani M, Shahab J, Garrakyarghi M, Khanjani R, Hedayatt P. Cobiniation of atorvastatin/coenzyme Q10 as adjunctive treatment in congestive heart failure: a double-blind randomized placebo-controlled clinical trial. ARYA Atheroscler. 2014;10(1):1-5
8. Trine Baur Opstad, Alexander J, Aaseth J, Larsson A, Ingebjørg Seljeflot, Urban Alehagen. Selenium and Coenzyme Q10 Intervention Prevents Telomere Attrition, with Association to Reduced Cardiovascular Mortality—Sub-Study of a Randomized Clinical Trial. Nutrients. 2022 Aug 15;14(16):3346–6.
9. Kumar A, Singh RB, Saxena M, Niaz MA, Joshi SR, Chattopadhyay P, et al. Effect of carni Q-gel (ubiquinol and carnitine) on cytokines in patients with heart failure in the Tishcon study. Acta Cardiologica. 2007 Jul 31;62(4):349–54.
10. Morisco C, Trimarco B, Condorelli M. Effect of coenzyme Q10 therapy in patients with congestive heart failure: a long-term multicenter randomized study. Clin Investigator. 1993;71(8 Suppl):S134–136
11. Kocharian A, Shabanian R, Rafiei-Khorgami M, Kiani A, Heidari-Bateni G. Coenzyme Q10 improves diastolic function in children with idiopathic dilated cardiomyopathy. Cardiology in the Young. 2009 Aug 25;19(5):501–6.
12. Kawashima C, Matsuzawa Y, Konishi M, Akiyama E, Suzuki H, Sato R, Nakahashi H, Kikuchi S, Kimura Y, Maejima N, et al. Ubiquinol improves endothelial function in patients with heart failure with reduced ejection fraction: a Single-Center, randomized double-blind placebo-controlled crossover pilot study. Am J Cardiovasc Drugs: Drugs Devices Other Interventions. 2020;20(4):363–72
13. Berman M, Erman A, Ben-Gal T, Dvir D, Georghiou GP, Stamler A, et al. Coenzyme Q10 in patients with end-stage heart failure awaiting cardiac transplantation: A randomized, placebo-controlled study. Clinical Cardiology. 2004 May;27(5):295–9.
14. Munkholm H, Hansen HH, Rasmussen K. Coenzyme Q10 treatment in serious heart failure. Biofactors. 1999;9(2–4):285–9
15. Keogh A, Fenton S, Leslie C, Aboyoun C, Macdonald P, Zhao YC, Bailey M, Rosenfeldt F. Randomised double-blind, placebo-controlled trial of coenzyme Q, therapy in class II and III systolic heart failure. Heart Lung Circ. 2003;12(3):135–41
16. Morisco C, Nappi A, Argenziano L, Sarno D, Fonatana D, Imbriaco M, Nicolai E, Romano M, Rosiello G, Cuocolo A. Noninvasive evaluation of cardiac hemodynamics during exercise in patients with chronic heart failure: effects of short-term coenzyme Q10 treatment. Mol Aspects Med. 1994;15:s155–163
17. Belardinelli R, Muçaj A, Lacalaprice F, Solenghi M, Seddaiu G, Principi F, Tiano L, Littarru GP. Coenzyme Q10 and exercise training in chronic heart failure. Eur Heart J. 2006;27(22):2675–81
18. Mareev VY, Minina YV, Mareev YV. Coenzyme Q-10 in treatment of patients with heart failure: results Russian multicenter double blind placebo controlled study. Eur J Heart Fail. 2017;19:56
19. Zhao Q, Kebbati AH, Zhang Y, Tang Y, Okello E, Huang C. Effect of coenzyme Q10 on the incidence of atrial fibrillation in patients with heart failure. J Invest Medicine: Official Publication Am Federation Clin Res. 2015;63(5):735–9
20. Bi J. Clinical Observation of Coenzyme Q10 on the efficacy of dilated cardiomyopathy patients with heart failure. Practical J Cardiac Cereb Pneumal Vascular Disease. 2012;20(11):1813–4
21. Permanetter B, Rössy W, Klein G, Weingartner F, Seidl KF, Blömer H. Ubiquinone (coenzyme Q10) in the long-term treatment of idiopathic dilated cardiomyopathy. Eur Heart J. 1992;13(11):1528–33
22. Hofman-Bang C, Rehnqvist N, Swedberg K, Wiklund I, Aström H. Coenzyme Q10 as an adjunctive in the treatment of chronic congestive heart failure. The Q10 Study Group. J Card Fail. 1995;1(2):101–7
23. Adarsh K, Kaur H, Mohan V. Coenzyme Q10 (CoQ10) in isolated diastolic heart failure in hypertrophic cardiomyopathy (HCM). Biofactors. 2008;32(1–4):145–9
24. Khatta M, Alexander BS, Krichten CM, Fisher ML, Freudenberger R, Robinson SW, Gottlieb SS. The effect of coenzyme Q10 in patients with congestive heart failure. Ann Intern Med. 2000;132(8):636–40
25. Watson PS, Scalia GM, Galbraith A, Burstow DJ, Bett N, Aroney CN. Lack of effect of coenzyme Q on left ventricular function in patients with congestive heart failure. J Am Coll Cardiol. 1999;33(6):1549–52
26. Wu Y. Coenzyme Q10 for the elderly with chronic heart failure efficacy and safety analysis. Chin J Geriatric Care. 2014;12(3):42–3
27. Feng Z, Bai T, Zhang L, Li X, Wang H, Wu R, Liu Y. Clinical Observation on 206 cases CHF treated with CoQ10 combined with routine therapy. Hebei Med. 2010;16(10):1184–7
28. Jiang Z. Clinical study of coenzyme Q10 combined with valsartan in treatment of chronic heart failure. Drugs Clin. 2019;34(2):346–50
29. Wu B, Su C, Liu Z, Chen P, Huang S, Li M, Xu J. Clinical observation of Qiliqiangxin capsule combined with coenzyme q10 in the treatment of chronic heart failure. J Guangdong Med Coll. 2019;37(6):662–5
30. Pei D, Zhao Q, Okello E, Yu S, Zhang Y. Effect of coenzyme Q10 administration on the incidence of atrial fibrillation in patients with chronic heart failure. Chin J Cardiac Pacing Electrophysiol. 2010;24(5):423–5
31. Wan X, Yang G, Zhou Q. Effect of trimetazidine combined with coenzyme Q10 on cardiac function in patients with chronic heart failure. Contemp Med. 2021;27(31):4–6
32. Zheng D, Lin C, Zhao X. Effects of Sacubitril Valsartan combined with Coenzyme Q10 in the treatment of patients with chronic heart failure. China J Pharm Econ. 2021;16(10):66–6871
33. Li N, Li H. The efficacy of coenzyme Q10 in the treatment of heart failure. J Practical Med. 1999;15(8):638
34. Yang D. Clinical observation of coenzyme Q10 in reducing the occurrence of major adverse cardiovascular events in patients with chronic heart failure. Chin J Clin Ration Drug Use. 2013;6(30):19–19
35. Ping G, Zidan L, Shufen L. Clinical observation of coenzyme Q10 in the treatment of heart failure. Mod Diagnosis Treat. 2015;26(13):2983–4
36. Liu Y. Effects of coenzyme Q10 combined with angiotensin II receptor antagonist on cardiac function and 6min walking distance in patients with chronic heart failure. J Hubei Univ Sci Technology(Medical Sciences). 2020;34(2):122–4
